# Supplementary material for: Central Sensitization Syndromes and Trauma: Mediating Role of Sleep Quality, Pain Catastrophizing, and Emotional Dysregulation Between Post-Traumatic Stress Disorder and Pain
Source: Healthcare (Basel). 2025 Sep 4;13(17):2221. doi: 10.3390/healthcare13172221 (PMC12428682; doi:10.3390/healthcare13172221)
Supplement: Supplementary file 1 [file healthcare-13-02221-s001.zip › healthcare-3789583-supplementary.pdf]

**Table S1.** Specific chronic pain syndromes in the central sensitization subgroup (*n* = 467)

| Chronic pain syndrome | *n* | % |
|---|---|---|
| Chronic back/neck pain | 227 | 48.6 |
| Migraine or tension headaches | 221 | 47.3 |
| Irritable bowel syndrome | 102 | 21.8 |
| Temporomandibular joint disorder | 48 | 10.3 |
| Fibromyalgia | 37 | 7.9 |
| Chronic pelvic pain | 37 | 7.9 |
| Chronic fatigue syndrome | 28 | 6 |
| Multiple chemical sensitivity | 11 | 2.4 |
| Comorbidity (≥ 2 CSS) | 258 | 55.25 |

**Table S2.** Medical conditions in the medical pathology group (*n* = 214)

| Medical conditions | *n* | % |
|---|---|---|
| Cardiac | 36 | 17.8 |
| Endocrine | 39 | 19.2 |
| Gynecological | 10 | 5.7 |
| Autoinmune | 22 | 11.3 |
| Respiratory | 27 | 13.6 |
| Traumatological | 36 | 17.8 |
| Neurological | 6 | 3.8 |
| Digestive | 9 | 5.2 |
| Dermatological | 7 | 6.3 |
| Nephrological | 3 | 2.4 |
| Oncological | 9 | 5.2 |
| Ophthalmological | 4 | 2 |
| Otorhinolaryngology | 3 | 1.5 |
| Comorbidity ( ≥ 2 conditions) | 33 | 15.4 |

**Table S3.** Prevalence (%) of traumatic events in the three study subgroups and the total sample

| | CSS ($n$ = 467) | MP ($n$ = 214) | Healthy ($n$ = 861) | Total sample ($N$ = 1542) | $\chi^2$ |
|---|---|---|---|---|---|
| Sudden or accidental death of a loved one | 33 | 34.1 | 23.7 | 28 | 23.28 *** |
| Transport accidents | 26.6 | 21 | 18.2 | 21.1 | 17.31 ** |
| Natural disasters | 27 | 18.2 | 17.8 | 20.6 | 19.01 ** |
| Bullying/psychological abuse in work/academic area | 26.3 | 19.2 | 15.8 | 19.5 | 30.90 *** |
| Bullying/psychological abuse in intrafamilial/partner area | 24.6 | 17.3 | 13.6 | 17.4 | 30.24 *** |
| Abuse sexual/rape by known/stranger people | 12 | 7 | 5.3 | 7.6 | 22.66 *** |
| Abuse sexual/rape by stranger people | 9 | 3.3 | 5.7 | 6.4 | 16.65 * |
| Other accidents | 18.6 | 12.6 | 11.6 | 13.9 | 17.26 ** |
| Serious illness | 12.4 | 17.3 | 6.2 | 9.6 | 37.65 *** |
| Intrafamilial/couple physical abuse | 16.3 | 8.9 | 5 | 8.9 | 61.42 *** |
| Physical violence in the community environment | 6.2 | 3.7 | 3.7 | 4.5 | 15.25 * |
| Repulsive situations | 3.2 | 1.4 | 2.9 | 2.8 | 8.73 |
| Imprisonment or detention | 3.2 | 1.4 | 1.6 | 2.1 | 10.68 |
| Combat or exposure to war zone | 1.5 | 0.5 | 1.2 | 1.2 | 9.01 |

| | | | | | |
|---|---|---|---|---|---|
| Terrorist acts, torture or kidnapping | 0.9 | 0.9 | 1 | 1 | 8.36 |
| Other traumatic event | 17.3 | 15 | 10.3 | 13.1 | 16.95 ** |

Note. CSSs = central sensitization síndromes, MP = medical pathologies. * $p < 0.05$; ** $p < 0.01$; *** $p < 0.001$.
